# Deciphering Potential Molecular Signatures to Differentiate Acute Myeloid Leukemia (AML) with *BCR::ABL1* from Chronic Myeloid Leukemia (CML) in Blast Crisis

**DOI:** 10.3390/ijms242015441

**Published:** 2023-10-22

**Authors:** Lara Boucher, Nathalie Sorel, Christophe Desterke, Mélanie Chollet, Laura Rozalska, Maria Pilar Gallego Hernanz, Emilie Cayssials, Anna Raimbault, Annelise Bennaceur-Griscelli, Ali G. Turhan, Jean-Claude Chomel

**Affiliations:** 1 CHU de Poitiers, Service de Cancérologie Biologique, F86000 Poitiers, France; lara.boucher23@gmail.com (L.B.); nathalie.sorel@chu-poitiers.fr (N.S.); anna.raimbault@chu-poitiers.fr (A.R.); 2 Faculté de Médecine, Université Paris Saclay, F94270 Le Kremlin-Bicêtre, France; christophe.desterke@gmail.com (C.D.); abenna@hotmail.fr (A.B.-G.); turviv33@gmail.com (A.G.T.); 3 CHU de Poitiers, Service d’Hématologie Biologique, F86000 Poitiers, France; melany_79@hotmail.fr (M.C.); laura.rozalska@chu-poitiers.fr (L.R.); 4 CHU de Poitiers, Service d’Oncologie Hématologique et Thérapie Cellulaire, F86000 Poitiers, France; maria-pilar.gallego-hernanz@chu-poitiers.fr (M.P.G.H.); emilie.cayssials@hotmail.fr (E.C.); 5 INSERM, CIC-P 1402, F86000 Poitiers, France; 6 INSERM U1310, F94807 Villejuif, France; 7 INGESTEM-ESTeam Paris Sud, F94800 Villejuif, France; 8 Service d’Onco-Hématologie, Hôpital Paul Brousse, AP-HP Université Paris Saclay, F94804 Villejuif, France; 9 Service d’Hématologie, Hôpital Bicêtre, AP-HP Université Paris Saclay, F94270 Le Kremlin-Bicêtre, France

**Keywords:** CML, AML, AML with *BCR::ABL1*, RNA-Seq, *CD25*, *ID4*

## Abstract

Acute myeloid leukemia (AML) with *BCR::ABL1* has recently been recognized as a distinct subtype in international classifications. Distinguishing it from myeloid blast crisis chronic myeloid leukemia (BC-CML) without evidence of a chronic phase (CP), remains challenging. We aimed to better characterize this entity by integrating clonal architecture analysis, mutational landscape assessment, and gene expression profiling. We analyzed a large retrospective cohort study including CML and AML patients. Two AML patients harboring a *BCR::ABL1* fusion were included in the study. We identified *BCR::ABL1* fusion as a primary event in one patient and a secondary one in the other. AML-specific variants were identified in both. Real-time RT-PCR experiments demonstrated that *CD25* mRNA is overexpressed in advanced-phase CML compared to AML. Unsupervised principal component analysis showed that AML harboring a *BCR::ABL1* fusion was clustered within AML. An AML vs. myeloid BC-CML differential expression signature was highlighted, and while *ID4* (inhibitor of DNA binding 4) mRNA appears undetectable in most myeloid BC-CML samples, low levels are detected in AML samples. Therefore, *CD25* and *ID4* mRNA expression might differentiate AML with *BCR::ABL1* from BC-CML and assign it to the AML group. A method for identifying this new WHO entity is then proposed. Finally, the hypothesis of AML with *BCR::ABL1* arising from driver mutations on a *BCR::ABL1* background behaving as a clonal hematopoiesis mutation is discussed. Validation of our data in larger cohorts and basic research are needed to better understand the molecular and cellular aspects of AML with a *BCR::ABL1* entity.

## 1. Introduction

The *BCR::ABL1* rearrangement, encoded by the Philadelphia (Ph1) chromosome and resulting from t(9;22)(q34;q11) translocation, is the molecular hallmark of chronic myeloid leukemia (CML), a well-known myeloproliferative neoplasm [1]. Historically, in the absence of adequate treatment, CML progresses inexorably from the chronic phase (CP) to the blast crisis (BC), also known as the acute phase, with or without an intermediate accelerated phase (AP). Recently, the reality of the AP has been questioned, and a more global concept of the advanced phases of CML has been proposed [2]. Today, with the use of ATP-competitive tyrosine kinase inhibitors (TKIs), (imatinib, dasatinib, nilotinib, bosutinib, and ponatinib) or an allosteric inhibitor (asciminib), the risk of CML transformation has significantly decreased, becoming a rare event, with an estimated annual progression rate to the advanced phases (AP or BC) of approximately 1% [3]. In most cases, blast crisis presents as acute myeloid leukemia (AML), less frequently as acute lymphoblastic leukemia (ALL), and rarely as mixed phenotype acute leukemia (MPAL) [1,4].

The *BCR::ABL1* rearrangement is also found in de novo AMLs, called AML with *BCR::ABL1* fusion or AML with t(9;22)(q34.1;q11.2) translocation in recent international classifications [2,5]. More than 20% of blasts are required to diagnose such acute leukemias. Furthermore, they need to be differentiated from the myeloid transformation of CML without an apparent CP. Regarding acute leukemias, the *BCR::ABL1* rearrangement can, therefore, be detected in AML arising from BC-CML, in de novo AML, and throughout the course of AML, as a secondary event. In this context, we believe that improved molecular analysis of AMLs harboring a *BCR::ABL1* rearrangement holds excellent promise for refining their classification and selecting the best therapeutic strategies.

High-throughput genomic DNA sequencing (NGS) has paved the way for accurate characterization of the mutational landscape of AMLs [6,7,8]. More recently, RNA sequencing (RNA-Seq) has enabled a more detailed diagnosis of AML, either by identifying fusion genes or by analyzing the transcriptome [9,10]. Nowadays, such approaches, based on targeted gene panels and applied in clinical routine, allow comprehensive and accurate investigation of AMLs.

CML stem cells (LSCs) are characterized by several surface markers, including CD25 (interleukin-2 receptor alpha chain), CD26 (dipeptidyl peptidase-4 or DPP4), and IL1-RAP (interleukin-1 receptor accessory protein) [11,12]. CD25 expression appears specific for CML LSCs compared to AML LSCs and normal stem cells [13]. We recently identified CD25 as a marker of CML progression using CML-derived induced pluripotent stem cells [14]. We showed that *CD25* mRNA expression was higher in BC-CML patients than in AML patients.

In this paper, we tried to determine whether AML with *BCR::ABL1* could be molecularly distinguished from acute transformations of CML and definitively assigned as typical AML. To this end, we used targeted NGS to determine the molecular landscape of AML harboring a *BCR::ABL1* fusion. We performed colony-forming cell (CFC) assays to understand the clonal architecture of these leukemias. We also examined *CD25*, *CD26*, and *IL1-RAP* mRNA expression to highlight a potential marker for BC-CML or AML. Finally, we analyzed the mRNA expression data obtained using RNA-Seq in an attempt to stratify patients according to their disease.

## 2. Results

### 2.1. Clinical and Biological Characteristics of BCR::ABL1-Positive AML Patients

AMLs carrying the *BCR::ABL1* rearrangement have often been described as de novo leukemias and, more rarely, as a secondary event during disease relapse. Two AML patients harboring a *BCR::ABL1* fusion were identified in our institution and extensively analyzed. The first patient (AML/BA-1) is representative of a de novo AML with *BCR::ABL1,* without CML history. As such, it can be distinguished from BC-CML. He was a 47 year old man with hyperleukocytosis (292 × 10^9^/L) and 75% peripheral blasts at diagnosis (Table 1). Bone marrow aspiration and flow cytometry diagnosed AML-FAB-M2. The ISTH (International Society on Thrombosis And Haemostasis) score based on four parameters (prothrombin time, platelets, fibrinogen, and fibrin-related markers) was consistent with overt DIC (disseminated intravascular coagulation). Cytogenetic analysis identified a t(9;22)(q34;q11) translocation resulting in a classical e14a2 *BCR::ABL1* rearrangement. No additional cytogenetic abnormality was observed. However, the *IDH2* and *NPM1* type A pathogenic variants were characterized using NGS. The patient started cytoreductive therapy (cytarabine + hydroxyurea) but died rapidly of multi-visceral failure associated with cerebral hematoma.

The second patient (AML/BA-2) represents an example of *BCR::ABL1* acquisition upon first AML relapse. She was a 61 year old woman with pancytopenia and 63.5% blasts in the peripheral blood at diagnosis. Bone marrow aspiration revealed massive infiltration by granular myeloblasts (92%), and flow cytometry confirmed the myeloid phenotype (Table 2).

The patient was subsequently diagnosed with AML-FAB-M1. A normal karyotype was found, and NGS identified three pathogenic variants in the *TET2* (two variants) and *NPM1* genes. The patient achieved complete cytologic remission after receiving the standard induction regimen (daunorubicin + cytarabine) and consolidation therapy (cytarabine). A hematologic relapse was observed 9 months after the disease onset, and a Philadelphia chromosome was detected. Molecular analyses characterized an atypical *BCR::ABL1* e6a2 rearrangement in addition to previously identified variants. Before allogeneic stem cell transplantation, the patient was treated with salvage chemotherapy (cytarabine, amsacrine, dasatinib). Five years later, the patient remains in molecular remission (*NPM1* and *BCR::ABL1* mRNA transcript follow-up).

### 2.2. Clonal Architecture and Tumor Evolution of BCR::ABL1-Positive AMLs

To determine the sequence of mutation acquisition at the hematopoietic stem cell (HSC)/progenitor level, CFC assays were performed using a bone marrow sample at diagnosis (patient AML/BA-1) or at relapse (patient AML/BA-2). At day +14, clonogenic cells (CFU-GM and CFU-G) were harvested from the methylcellulose. *BCR::ABL1* fusion and specific mutations were researched in each CFC using qRT-PCR. The clonal evolution of the disease was then described using CFC assays and NGS/RNA-Seq data.

**Figure 1:**
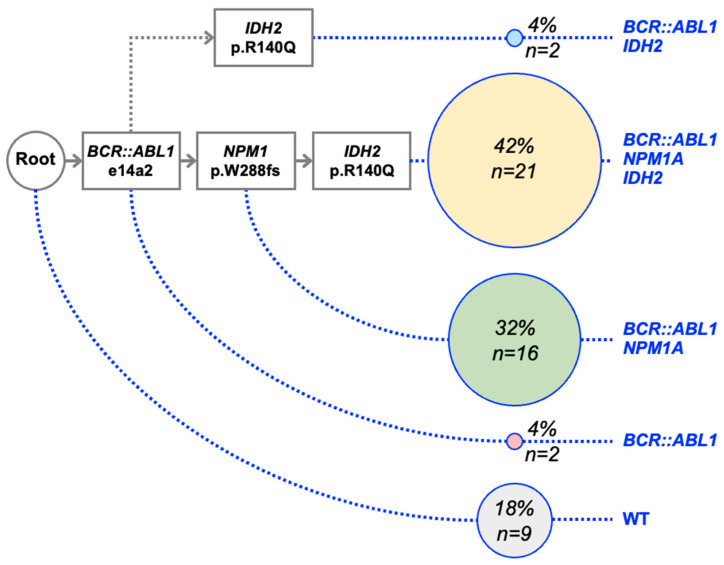
Clonal architecture and mutational evolution of patient AML/BA-1. The presence of e14a2 *BCR::ABL1* fusion and *IDH2* and *NPM1* variants was assessed using qRT-PCR in 50 individual CFCs from the bone marrow sample at diagnosis. The number of CFCs harboring a specific genetic pattern was indicated. This protocol highlighted a *BCR::ABL1*—*NPM1A*—*IDH2* major sequence.

For the AML/BA-1 patient, the presence of *BCR::ABL1* fusion and the *IDH2*/*NPM1* variants was evaluated from 50 individual CFCs. These experiments revealed a major *BCR::ABL1*-*NPM1*-*IDH2* sequence and a minor *BCR::ABL1*-*IDH2* one (Figure 1). Most HSCs or progenitors carry at least the *BCR::ABL1* fusion and the *NPM1* mutation. Using these data, it was possible to apprehend the tumoral evolution of the de novo AML at the time of diagnosis. In this case, *BCR::ABL1* fusion acts as a primary event, and the other mutations (*NPM1* and *IDH2*) as secondary cooperative events.

Clonogenic assays were also used to understand the clonal architecture from the bone marrow NGS or RNA-Seq results. In contrast to the CFCs, the bone marrow bulk cells presented all three genetic abnormalities with equal frequency (Table 3).

**Figure 2:**
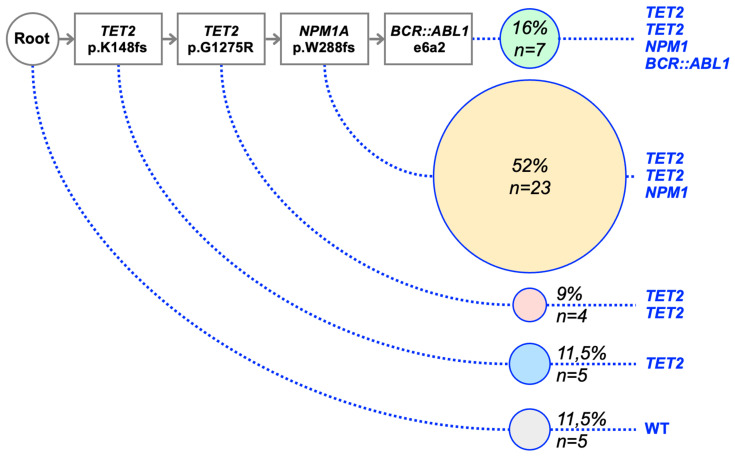
Clonal architecture and mutational evolution of patient AML/BA-2. Real-time RT-PCR determined the presence of *TET2* and *NPM1* variants along with e6a2 *BCR::ABL1* rearrangement in 44 individual CFCs from the bone marrow sample at relapse. The number of CFCs harboring a specific genetic pattern was indicated. This procedure highlighted a *TET2* (K148fs)—*TET2* (G1275R)—*NPM1A*—*BCR::ABL1* sequence.

For patient AML/BA-2, the presence of the *TET2*/*NPM1* variants and *BCR::ABL1* fusion was estimated from 44 individual CFCs representative of the relapse. Clonogenic assays highlighted a *TET2* (K148fs)-*TET2* (G1275R)-*NPM1*-*BCR::ABL1* sequence (Figure 2). It should be noted that only a few hematopoietic stem/progenitor cells presented the complete sequence. In this case, *BCR::ABL1* fusion operated as a secondary event (proliferating mutation), as expected. NGS and RNA-Seq analyses should also be interpreted based on the clonal architecture determined by the CFC assay.

As shown in Table 4, the NGS and RNA-Seq performed on the bone marrow bulk identified all four abnormalities with comparable frequency, suggesting a massive expansion of *BCR::ABL1-*expressing cells.

The major clonal characteristics of the AML/BA-1 and AML/BA-2 patients are summarized in Table 5. The clonal architecture established at the progenitor/stem cell level identifies AML/BA-1 as a true AML with *BCR::ABL1*.

### 2.3. CD25, CD26, and IL1-RAP mRNA Expression in AML and Advanced-Phase CML

We previously showed that *CD25* mRNA expression was higher in advanced-phase CML than in CP-CML and AML [14]. Here, we tested *CD25*, *CD26*, and *IL1-RAP* mRNA expressions as potential biomarkers to distinguish advanced-phase CML from AML. Three sub-cohorts, including 26 AML patients, 33 CP-CML patients, and 15 AP/BC-CML patients, were analyzed. It should be noted that the advanced phases of CML include AP-CML and BC-CML, as detailed in Table S1. To avoid bias in the results, the two patients with *BCR-ABL1*-positive AML were not included in the analysis.

**Figure 3:**
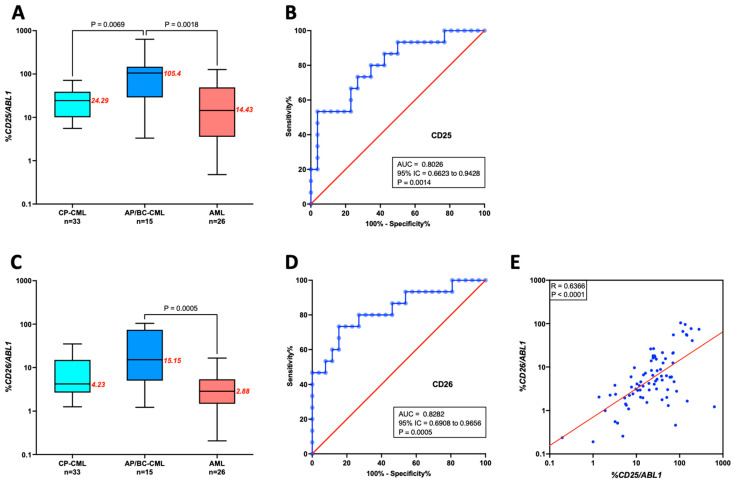
*CD25* and *CD26* mRNA expression in CML and AML patients. (**A**,**C**). The graphs show the *CD25* and *CD26* mRNA expressions in CML patients in chronic phase at diagnosis (CP-CML), in patients in accelerated phase (AP) or blast crisis (BC), and in patients with AML. Data were shown as box-and-whisker plots (5–95 percentile). The median is shown in red for each group, and a two-tailed Mann–Whitney test calculated the *p*-value. (**B**,**D**). ROC curves suggest that *CD25* and *CD26* mRNA expression can distinguish AP/BC patients (n = 15) from AML controls (n = 26). (**E**). Correlation between *CD26* and *CD25* mRNA expression.

As previously reported, *CD25* mRNA was significantly overexpressed in advanced-phase CML compared to CP-CML and AML (Figure 3A). It seems important to note that those results were obtained using independent patient cohorts from our first study [14]. In addition, an analysis of the ROC curve suggests that *CD25* mRNA expression behaves as a good marker of advanced-phase CML with an AUC > 0.8 (Figure 3B). Comparable results were obtained for *CD26* mRNA expression (Figure 3C,D). In contrast, *IL1-RAP* mRNA expression did not vary significantly between the three cohorts. As a result, *CD25* or *CD26* mRNA expression may be relevant indicators in differentiating AML from advanced-phase CML, allowing for better characterization of *BCR::ABL1*-positive AML. Moreover, a positive correlation was observed between *CD26* and *CD25* mRNA expression (Figure 3E). Consequently, only *CD25* mRNA expression was used for the rest of the work.

### 2.4. Unsupervised Analyses of Gene Expression from RNA-Seq Data

The RNA-Seq expression data from AML and CML patients already analyzed for fusions and variant detection were extracted and subsequently analyzed (Table S2). These data included 30 AML samples (18 de novo AML and 12 secondary AML or sAML) and 20 CML samples (six patients with BC-CML, five with resistant CP-CML, and nine with CP-CML at diagnosis). To be consistent with our objective, only the myeloid BC-CML (M-BC-CML) samples were retained. Six PB samples from healthy donors and the two patients to be tested, AML/BA-1 (PB and BM at diagnosis) and AML/BA-2 (BM at diagnosis and relapse), were also analyzed. Three genes were discarded due to insufficient reads (*MLLT4* and *U2AF1*) or a lack of expression in all samples (*ROS1*).

**Figure 4:**
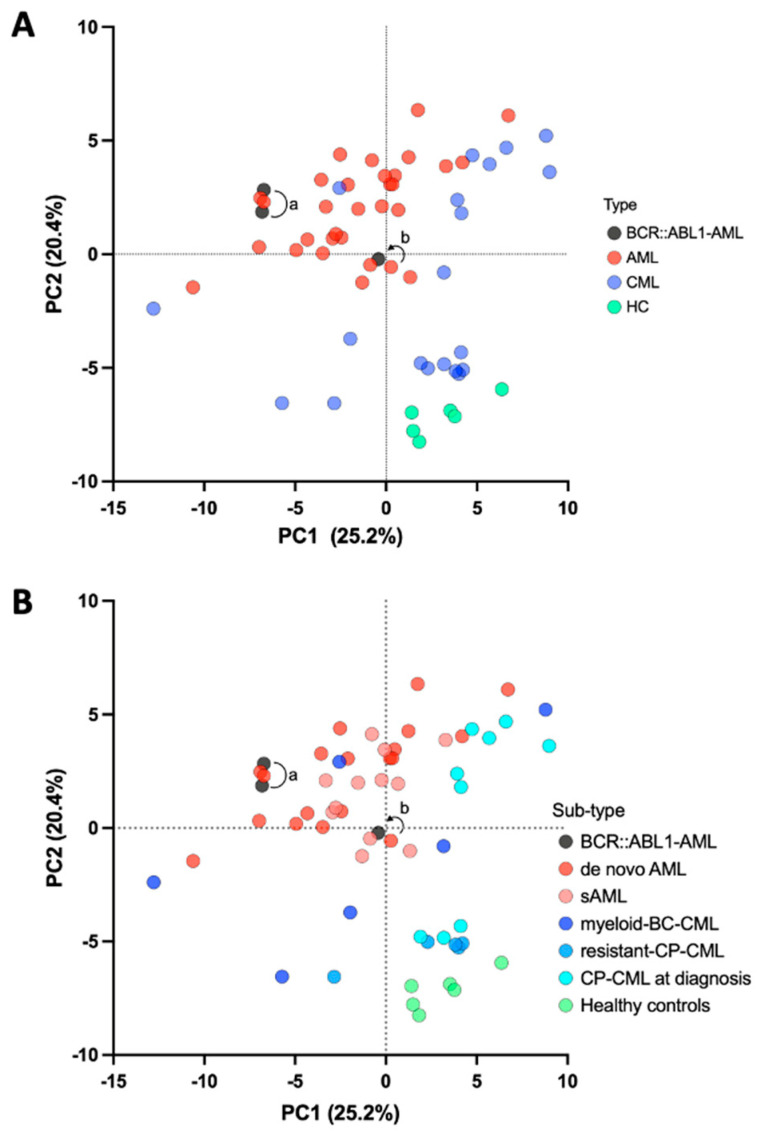
Unsupervised principal component analysis (PCA) on RNA-Seq data. (**A**). Unsupervised PCA was performed across AML, CML, healthy controls, and *BCR::ABL1*-AML types. (**B**). The same investigations were also based on sample subtypes (AML, sAML, CP-CML at diagnosis, resistant CP-CML, M-BC-CML, healthy controls, and *BCR::ABL1*-AML). In the figure, (a) represents the peripheral blood and bone marrow samples from patient AML/BA-1 (black circles), and (b) shows the bone marrow samples from patient AML/BA-2 at diagnosis (red circle) and relapse (black circle).

The transcriptome-normalized matrix was processed using principal component analysis for unsupervised analyses. These studies revealed a clear stratification of the healthy donor controls, CML, and AML samples (Figure 4A). *BCR::ABL1*-positive AML samples were found to be clustered within the AML samples (a, AML/BA-1 and b, AML/BA-2 at relapse). Regarding the AML/BA-2 patient, the progression from de novo AML (b, without *BCR::ABL1*, red circle) to relapse (b, with *BCR::ABL1*, black circle) occurred without any significant changes in the gene expression profile. The same investigations based on the sample subtypes allowed for good stratification of the CML samples (CP-CML at diagnosis, resistant CP-CML, and myeloid BC-CML) (Figure 4B). Conversely, these transcriptome analyses did not stratify secondary AML and de novo AML.

### 2.5. AML vs. Myeloid BC-CML Differential Expression Signature

**Figure 5:**
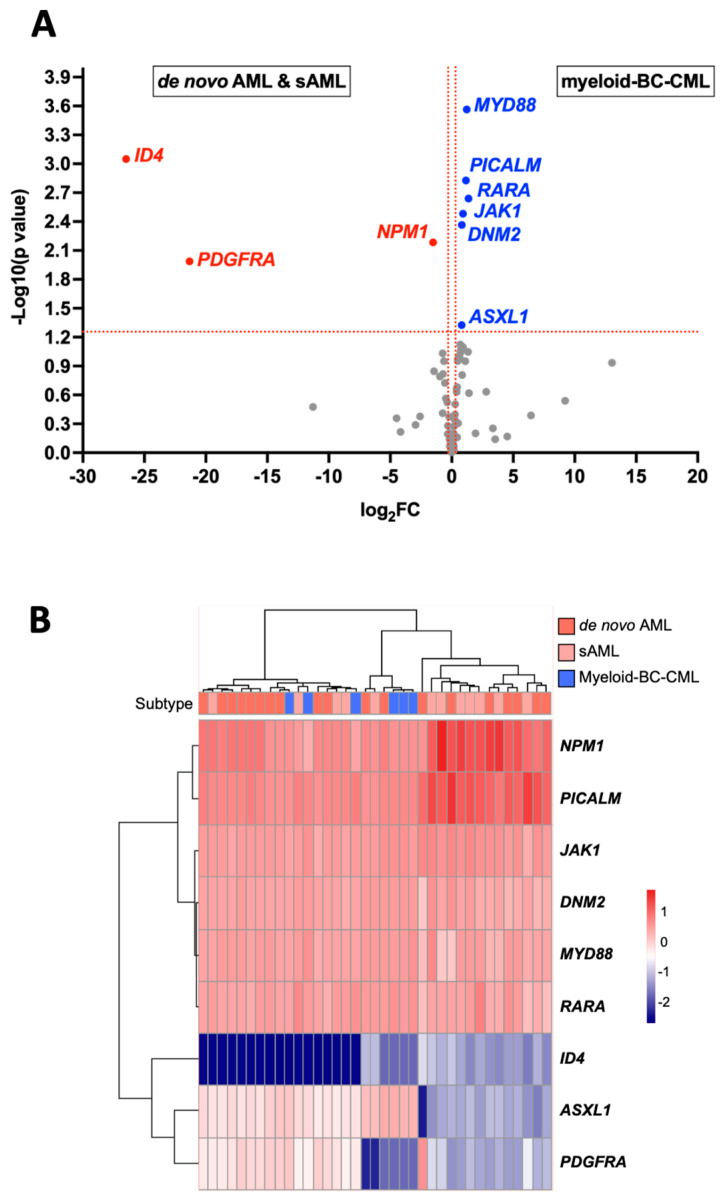
Genes differentially expressed between myeloid BC-CML and AML samples. (**A**). The volcano plot highlights genes overexpressed in the AML or BC-CML context. Scattered points represent genes, the x-axis is the log2 fold change, and the y-axis represents the −log10 (*p*-values). (**B**). Heatmap of differentially expressed genes. The list of significantly differentially expressed genes (*p*-value < 0.05) is shown. BC-CML samples are reclassified into the left clusters of the heatmap.

The transcriptome-normalized matrix was used for supervised analysis, highlighting the genes differentially expressed between the myeloid blast crisis CML and AML samples (Figure 5A). After false discovery rate adjustment of the *p*-values, nine genes appear to be differentially expressed between AML and M-BC-CML. In particular, *MYD88*, *PICALM*, and *RARA* are overexpressed in M-BC-CML with log2 fold changes of 1.22, 1.16, and 1.37, respectively (Table S3). On the other hand, *ID4* is overexpressed in AML samples with a log2 fold change of 26.5. With a cutoff of a raw *p*-value ≤ 0.05, it was possible to identify an expression profile that tended to reclassify BC-CML samples into the left clusters of the heatmap after clustering by the Euclidean distances (Figure 5B). These results suggest that the myeloid BC-CML samples had a different expression profile from the AML samples. Moreover, *ID4* (inhibitor of DNA binding 4) is a very attractive marker for distinguishing BC-CML from AML.

### 2.6. BCR::ABL1-Positive AML Characteristic Profile

**Figure 6:**
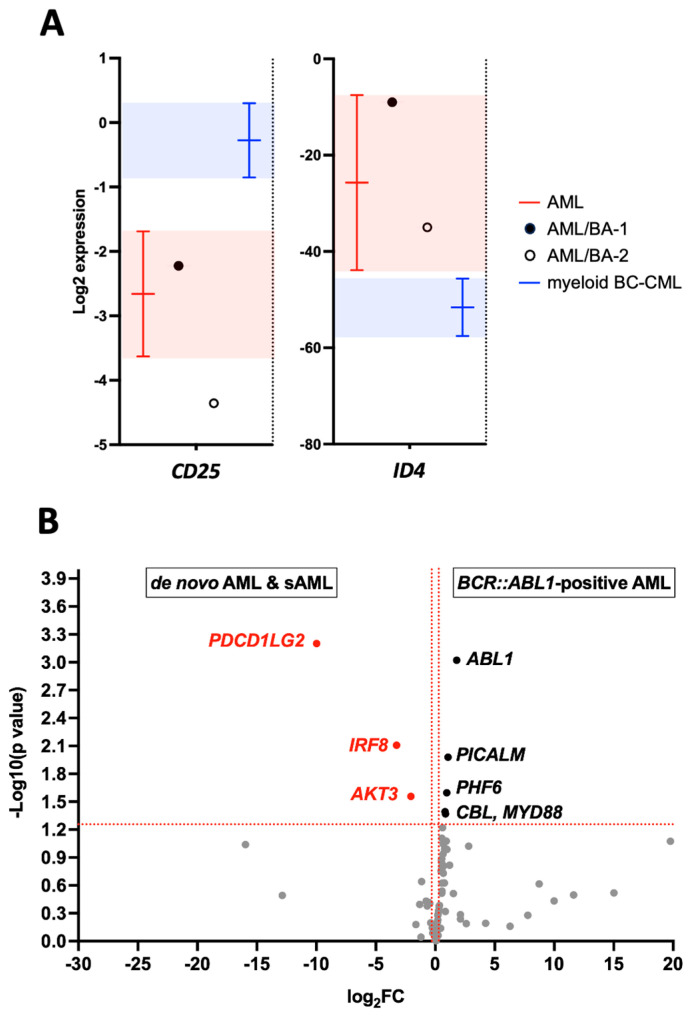
Molecular features of *BCR::ABL1*-expressing AML samples. (**A**). The graph shows *ID4* and *CD25* mRNA expressions in AML and M-BC-CML represented by the mean with a 95% confidence interval, highlighted in red and blue for AML and M-BC-CML, respectively. This representation indicates AML/BA-1 and AML/BA-2 samples as plain or empty black circles. AMLs harboring the *BCR::ABL1* fusion are separated from BC-CML and segregated with AML. (**B**). The volcano plot highlights genes overexpressed in *BCR::ABL1*-positive or -negative AML context. Scattered points represent genes, the x-axis is the log2 fold change, and the y-axis represents the −log10 (*p*-values).

This study identified two molecular markers: *ID4* using RNA-Seq and *CD25* (or *CD26*) using qRT-PCR. To verify their efficacy in distinguishing *BCR::ABL1* AMLs, we performed *CD25* mRNA quantification on the entire RNAseq sub-cohort. The results for the *CD25* and *ID4* expression in the AML and M-BC-CML samples are presented in Figure 6A as the mean with a 95% confidence interval. The expression data from the AML/BA-1 and AML/BA-2 patients (circles in the graph) fall within the AML confidence interval.

Lastly, differential expression analyses were performed between the *BCR::ABL1*-positive AML and other AML samples (Figure 6B). After false discovery rate adjustment of the *p*-values, a significant over-expression of *ABL1* in the *BCR::ABL1*-positive AML as compared to the other AML samples (log2 Fold Change = +1.80, adjusted *p*-value = 0.039) is observed (Table S4). This result is consistent with the expression of the fusion transcript (assessed by the *ABL1* expression) in the *BCR::ABL1*-positive AML subtype. We also show a significant down-regulation of *PDCD1LG2* in *BCR::ABL1*-expressing AML (log2 Fold Change = −9.97, adjusted *p*-value = 0.039). *PDCD1LG2* (programmed cell death 1 ligand 2) encodes an immune checkpoint receptor ligand. It should be noted that this gene was not differentially expressed between the BC-CML and AML samples.

## 3. Discussion

New AML classifications have validated “AML with *BCR::ABL1*”, also called “AML with t(9;22)(q34.1;q11.2)”, as a distinct entity [2,5]. The diagnosis of AML with *BCR::ABL1* could be established without evidence of CML history. Compared to myeloid BC-CML, AML with *BCR::ABL1* seemed to be characterized by the absence of basophilia and a karyotype with less than 100% Ph1 chromosomes [15]. However, these data did not clearly distinguish AML with *BCR::ABL1* from M-BC-CML.

Although other mutations may play a role in disease prognosis, the *BCR::ABL1* oncogene has long been known to be the trigger for CML [16,17]. AMLs, on the other hand, require a known and complex multi-step process that ultimately leads to a blockage of the hematopoietic differentiation and the uncontrolled proliferation of immature leukemic cells [7,18]. Acquisition of a *BCR::ABL1* rearrangement during AML evolution is rare [19]. In this context, secondary *BCR::ABL1* fusion could act as a late cooperating event within the multi-step pathogenesis. It may also arise from de novo AML, in which *CBFB::MYH11*, *GATA2::MECOM*, *KMT2A::AFDN*, *PML::RARA*, or *RUNX1::RUNX1T1* are rearranged at diagnosis [20,21,22,23,24,25,26,27]. Secondary *BCR::ABL1* fusion has also been found to occur in AML after treatment with an FLT3 inhibitor [28,29]. Not all these cases meet the definition of AML with *BCR::ABL1* since *BCR::ABL1* acts as a secondary event and is likely to confer a proliferative advantage, such as *NRAS*, *FLT3*, or *KIT* mutations. Therefore, it appears essential to establish whether *BCR::ABL1* is a primary or secondary event through the multi-step sequence of AML. Consequently, to identify a putative AML with *BCR::ABL*, it seems necessary not only (1) to verify that *BCR::ABL1* is the first event of the multi-step process (using clonogenic assay and single-cell analysis), (2) to highlight the presence of typical AML abnormalities in addition to *BCR::ABL1* (using NGS and RNA-Seq), but also (3) to eliminate “specific” BC-CML mutations, such as ABL1 TKD mutations.

One of the major issues is whether or not AMLs with *BCR::ABL1* share a characteristic mutational landscape. Various approaches are available today to answer these questions, from older methods such as fragment analysis or Sanger sequencing to more global methods such as NGS or RNA-Seq. Overall, the AML driver genes include (according to frequency) *FLT3*, *NPM1*, *DNMT3A*, *NRAS*, *IDH1*/*2*, *RUNX1*, *TET2*, *WT1*, *ASXL1*, *PTPN11*, *SRSF2*, *TP53*, *CEBPA*, *BCOR*, *KMT2A*, and *KRAS* [30]. However, these data need to be qualified because AMLs are heterogenous malignancies. For example, the mutational landscape may be specific to cytogenic groups, resulting in molecular subgroups [7,31]. It may also differ between de novo AML and sAML or t-AML (therapy-related AML) [32].

Several studies focusing on M-BC-CML, particularly those based on high-throughput sequencing approaches, have been published in the last decade [33,34,35,36,37]. They showed that in most cases, at least one pathogenic mutation (in addition to *BCR::ABL1*) is detected during the progression to the blast phase. Pathogenic variants of *RUNX1*, *ASXL1*, and BCR::ABL1 TKD mutations (involved in TKI resistance) are frequently found. *BCR::ABL1* mutations are a well-known characteristic feature of TKI-resistant CMLs. *RUNX1* mutations may not be a specific marker for M-BC-CML, as they are also found in AMLs (albeit less frequently). Interestingly, *NPM1* mutations (common in AML) appear to be absent or extremely rare in BC-CML [38,39,40,41]. Considering all these data, can we characterize AMLs with *BCR::ABL1* by their mutational landscape? Recent studies based on NGS experiments have shown that the genetic profiles of AMLs harboring a *BCR::ABL1* fusion are not significantly different from those of M-BC-CML [35,42]. Therefore, it appears that only a few genetic markers other than the potential presence of an *NPM1* variant and the absence of a BCR::ABL1-TKD mutation can distinguish Ph1-positive AMLs from M-BC-CMLs and link them to Ph1-negative AMLs [43].

Some cell surface markers have been shown to distinguish CML LSCs from AML LSCs (CD34^+^CD38^−^) using multicolor flow cytometry [13,44]. In contrast to AML LSCs, CML LSCs can display a characteristic expression profile including CD25 (interleukin-2 receptor alpha chain), CD26 (dipeptidyl peptidase-4), or IL1-RAP (interleukin-1 receptor accessory protein) [44]. It has been proposed that CD25 and CD26 may be accurate markers of CML LSCs and potential therapeutic targets [11,13]. Recently, we established a BC-CML model using several CML-derived iPSC (induced pluripotent stem cell) lines mutagenized with N-ethyl-N-nitrosourea (an alkylating agent) [14]. Mutagenized CML iPSCs (representing BC-CML) and non-mutagenized CML iPSCs (representing CP-CML) were compared at the transcriptomic level after hematopoietic differentiation. Several genes were found to be upregulated in BC-CML, including *CD25*. These findings were confirmed in patients with CP-CML, BC-CML, AML, B-ALL (B-acute lymphoblastic leukemia), and healthy controls. *CD25* mRNA transcripts were found to be over-expressed in BC-CML in comparison to all other categories. Based on independent patient cohorts, these data were corroborated in the present study, highlighting their relevance. Notably, *CD25* mRNA was shown to be expressed in all samples and significantly overexpressed in BC-CML. All in all, *CD25* mRNA expression may be an attractive marker to differentiate BC-CML from AML.

In our RNA-Seq analyses, *ID4* mRNA appears undetectable in most M-BC-CML samples, while low levels are detected in AML samples. ID4 is critical for embryogenesis and fetal development and may also be involved in tumorigenesis [45]. ID4 may play seemingly contradictory roles in cancer cells. It could be over- or under-expressed at the RNA level, and the DNA promoter could be hypermethylated [46]. It has been reported that *ID4* expression silencing using promoter methylation is increased during CP to BC progression in CML [47]. The mRNA expression of *PDCD1LG2*, also called *PD-L2*, *CD273*, or *B7-DC*, was shown to be increased in a small proportion of bone marrow CD34+ or blood mononuclear cells from AML patients [48]. In this study, we have shown that the *PDCD1LG2* mRNA was under-expressed in *BCR::ABL1*-positive AML compared to de novo and secondary AML. Its impact on the disease and its potential interest in identifying AML harboring the *BCR::ABL1* fusion is unknown.

Our work shows that a comprehensive molecular analysis might be necessary to characterize leukemias more likely to correspond to AMLs with the *BCR::ABL1* WHO (World Health Organization) entity (Table 6). Identification of *BCR::ABL1* as the primary genetic event is critical, as are the presence of AML-specific secondary abnormalities (especially *NPM1* mutations) and the absence of ABL1 TKD point mutations. *CD25* and *ID4* mRNA expression may also be helpful, but this needs to be confirmed in larger patient cohorts.

Overall, our results support the inclusion of AML with *BCR::ABL1* within the AML category. However, the identification of the *BCR::ABL1* fusion (considered the hallmark of CML) as the primary oncogenic event through the clonal ontogeny of these cases of acute leukemia raises several concerns. *BCR::ABL1* rearrangement has been sporadically detected at low levels in the blood of healthy individuals [49,50,51,52]. Several hypotheses could explain why they do not develop CML despite the presence of the *BCR::ABL1* oncogene. Some studies have suggested that *BCR::ABL1* expression alone (or low-level *BCR::ABL1* expression) may not be sufficient to trigger CML leukemogenesis [53,54,55,56]. *BCR::ABL1* fusion could occur within a committed progenitor that has lost its stem cell properties [52]. It is now well established that leukemogenesis is characterized by its progressive nature, molecular diversification, and the clonal evolution of founder cells [57,58,59]. Six stages of cancer development were recently proposed, ranging from early phases to overt malignancy [60]. Concerning pre-malignant conditions, several molecular abnormalities detectable in healthy individuals can be characterized. Passenger or low-level driver mutations would correspond to CHIP (clonal hematopoiesis of indeterminate potential) or CHOP (clonal hematopoiesis with oncogenic potential) conditions. In these circumstances, *BCR::ABL1* could be considered a CHOP mutation likely to progress to overt leukemia subsequent to a variable latency period.

Based on these data, we can formulate a hypothesis worthy of discussion. In AMLs with *BCR::ABL1*, we can speculate that the *BCR::ABL1* fusion (primitive event) would act as a clonal hematopoiesis mutation. The subsequent acquisition of leukemogenesis-driving abnormalities such as *NPM1* or *IDH1/2* mutations could lead to the sudden development of de novo AML without passing through CP-CML. While this hypothesis appears attractive, it nevertheless requires further validation.

In conclusion, our study provides some new molecular clues helping to characterize AML with *BCR::ABL1*. Distinguishing these AMLs from myeloid BC-CMLs remains challenging, and fundamental research is required to better understand the molecular and cellular specificity of AMLs with *BCR::ABL1*. Given the rarity of these AMLs, a multicenter study is needed to analyze a significant number of patients. It would also be helpful to consider a single-cell analysis approach to assess the clonal architecture. In addition, this work highlights the importance of gene expression signatures. In this context, a more global transcriptomic approach could be an attractive perspective for better characterization of AMLs with *BCR::ABL1*.

## 4. Materials and Methods

### 4.1. Patients

In this study, we analyzed bone marrow or peripheral blood samples from 63 CML patients (19 advanced-phase CML, 5 TKI-resistant CP-CML, 39 and CP-CML at diagnosis) and 54 AML patients (37 de novo AML and 17 secondary AML or sAML). Molecular and cellular experiments (NGS, RNA-Seq, qRT-PCR, and clonogenic assays) were performed on residual sample material (leftovers after routine analysis). Among all patients, sub-cohort studies provided expression data using RNA-Seq or RT-qPCR (*CD25*, *CD26* or *IL1-RAP*). Two patients with AML and *BCR::ABL1* rearrangements (AML/BA-1 and AML/BA-2) had their bone marrow and peripheral blood extensively analyzed. The details about the patients (disease, karyotype, molecular rearrangements, pathogenic variants, and the availability of the RNA-Seq and qRT-PCR experiments) are provided in Table S5. A cohort of 6 healthy donors was used as the control. The study was carried out in accordance with the Declaration of Helsinki.

### 4.2. Targeted High-Throughput DNA and RNA Sequencing

Myeloid-targeted NGS was used to characterize pathogenic variants with their VAFs (variant allele frequencies). The NGS analyses were performed using the Agilent SureSelect Target Enrichment System and a personalized myeloid panel of 85 genes (Agilent Technologies, Santa Clara, CA, USA). An in-house bioinformatics pipeline was used to analyze the raw data after sequencing. RNA-Seq was used to characterize the fusion transcripts, to identify some pathogenic variants with their TAFs (transcript allele frequencies), and to estimate the mRNA expression of the targeted genes (expressed as log2 expressions using *CHMP2A*, *GPI*, *RAB7A*, and *VCP* as housekeeping genes). RNA-Seq was performed using the Archer^®^ FusionPlex^®^ Myeloid kit with a target set of 84 genes (Archer DX, Boulder, CA, USA). After sequencing, the raw data were analyzed using the Archer Analysis bioinformatics system. All libraries (DNA/RNA) were sequenced on the Illumina MiSeq platform (Illumina, San Diego, CA, USA).

### 4.3. Clonogenic Assays

CFC assays were used to characterize the sequence of genetic events in AML patients carrying a *BCR::ABL1* rearrangement. The CFC assays were performed on bone marrow samples at diagnosis (patient AML/BA-1) or relapse (patient AML/BA-2) as previously reported [61]. At day +14, clonogenic cells (mainly CFU-GM and CFU-G) were plucked from the methylcellulose (STEMCELL Technologies, Vancouver, Canada), and the total RNA was extracted from each colony. Real-time RT-PCR was carried out using a specific set of primers and probes (Table S6). In all cases, *ABL1* was used as an internal control to discard the samples where RNA extraction had failed. The experiment aimed to classify the clones according to their distinct genetic expression profiles.

### 4.4. CD25, CD26, IL1RAP mRNA Expression

The total RNA from the whole blood or bone marrow samples was reverse-transcribed using the High-Capacity cDNA Reverse Transcription Kit (Life Technologies, Foster City, CA, USA), and the qRT-PCR experiments were performed using the StepOnePlus Real-Time PCR System (Life Technologies, Foster City, CA, USA). The primers and TaqMan probes for *CD25*, *CD26*, *IL1-RAP*, and *ABL1* are described in Table S6. The percentages of *CD25*/*ABL1*, *CD26*/*ABL1*, and *IL1-RAP*/*ABL1* were determined using the ΔΔCt method using *ABL1* as a housekeeping gene, and a calibrator sample was analyzed in each experiment to ensure good reproducibility of the assays [62].

### 4.5. Graph and Statistical Analysis

The scatter plots, box-and-whisker charts, and receiver operating characteristic (ROC) curves were generated using GraphPad Prism 9 for macOS (GraphPad Software, San Diego, CA, USA, www.graphpad.com). Sample logistic regression, total area under the curve (AUC), and statistical analyses were performed using GraphPad Prism. The statistical parameters, including the statistical significance (*p*-value), are reported in the figures, and a *p*-value  <  0.05 was used as the cutoff.

### 4.6. RNA-Seq Transcriptome Analysis

The bioinformatic analyses performed on the normalized complete transcriptome matrix were carried out with “transpipe” R package version 1.4.0 (available at https://github.com/cdesterke/transpipe14, accessed on 11 September 2023) in the R software 4.2.1 environment. Principal component analysis (PCA) was carried out using the “pcatrans” function from the R package transpipe and depended on the “prcomp” R-based function and on the “autoplot” function from the ggfortify R package. Differentially expressed gene analyses were carried out with the “deg” function from the transpipe R package, which depends on the limma algorithm [63]. The volcano plots were drawn based on the limma results using the “vollimma” function from the transpipe R package, and the expression heatmap (Euclidean distances) was drawn using the “bestheat” function from the “transpipe” R package, which depends on the “pheatmap” R package.

## Figures and Tables

**Table 1:** Patient AML/BA-1 (de novo AML with *BCR::ABL1*), biological parameters at diagnosis.

	At Diagnosis	
	Peripheral blood	Bone marrow
Age (years)	46.7	
WBC (×10^9^/L)	292.4	
Neutrophils (×10^9^/L)	32.16	
Basophils (×10^9^/L)	2.92	
RBC (×10^12^/L)	4.22	
Hemoglobin (g/dL)	12.5	
Hematocrit (%)	37.8	
Platelets (×10^9^/L)	29	
Fibrin monomer (mg/mL)	137	
Blasts (%)	75	63
Flow cytometry		cMPO^−^, cCD79a^−^, cCD3^−^, CD34^−^, DR^−^, CD117^−^, CD13^+^, CD33^+low^, CD38^+^, CD19^−^, CD10^−^, CD4^−^, CD7^−^, CD11b^+low^, CD14^−^, CD64^−^
Conventional cytogenetics		46, XY, t(9;22)(q34;q11) [17]
RT-PCR analysis	*BCR::ABL1* (e14a2)	
NGS	*IDH2* p.R140Q (c.419G>A), *NPM1* p.Trp288CysfsTer12 (c.863_864insTCTG)	

WBC, white blood cells; RBC, red blood cells.

**Table 2:** Patient AML/BA-2 (*BCR::ABL1* acquisition at relapse), biological parameters at diagnosis and relapse.

	At Diagnosis	At Relapse (Diagnosis + 9.5 Months)
Age (years)	60.5	61.3
WBC (×10^9^/L)	10.4	6.1
Neutrophils (×10^9^/L)	0.44	3.76
Basophils (×10^9^/L)	0.06	0.03
RBC (×10^12^/L)	2.86	4.98
Hemoglobin (g/dL)	9.1	13.5
Hematocrit (%)	27.7	40.9
Platelets (×10^9^/L)	140	215
Blasts in PB (%)	63.5	11.6
Blasts in BM (%)	92	54
Flow cytometry (BM)	cMPO^+^, CD33^+^, CD117^+^, CD34^−^, CD56^low^, CD7^+^	CD33^+^, CD117^+^, CD34^−^, CD56^high^, CD7^−^
Conventional cytogenetics	46, XX [32] (BM)	46, XX, t(9;22)(q34;q11) [25]/46, XX [1] (PB)
FISH analysis (*BCR::ABL1* fusion)	Negative (BM) * 35 metaphase chromosomes and 200 interphase cells analyzed	Positive (PB), 80% 200 interphase cells analyzed
RT-PCR analysis (BM) (*BCR::ABL1* fusion)	Negative *	*BCR::ABL1* (e6a2)
NGS (BM)	*NPM1* p.Trp288CysfsTer12 (c.863_864insTCTA), *TET2* p.Lys148AsnfsTer3 (c.444_447delAGAA), *TET2* p.Gly1275Arg (c.3823G>A)	

WBC, white blood cells; RBC, red blood cells; PB, peripheral blood; BM, bone marrow; FISH, fluorescence in situ hybridization; * analyzed retrospectively.

**Table 3:** Results of NGS and RNA-Seq experiments for patient AML/BA-1.

Gene ID	Variant/Fusion	NGS (% of Reads)	RNA-Seq (% of Reads)
*BCR::ABL1*	*BCR::ABL1* e14a2		44.4
*IDH2*	p.Arg140Gln	29.9	45.8
*NPM1*	p.Trp288CysfsTer12	23.5	49.4

**Table 4:** Results of NGS and RNA-Seq experiments for patient AML/BA-2.

		At Diagnosis		At Relapse	
Gene ID	Variant/Fusion	NGS (% of Reads)	RNA-Seq (% of Reads)	NGS (% of Reads)	RNA-Seq (% of Reads)
*TET2* (1)	p.Lys148AsnfsTer3	46		29.5	
*TET2* (2)	p.Agly1275Arg	45.3		23.5	
*NPM1*	p.Trp288CysfsTer12	45.3	15.7	18.1	16.5
*BCR::ABL1*	*BCR::ABL1* e6a2		Not detected		17.7

**Table 5:** Clonal characteristics of AML/BA-1 and AML/BA-2 patients. CFCs, colony-forming cells.

	AML/BA-1	AML/BA-2
*BCR::ABL1* rearrangement	Primary event	Secondary event
AML-specific mutations (e.g., *NPM1*)	Secondary event	Primary event
% of *BCR::ABL1*-expressing CFCs	100%	16%
Conclusion	De novo AML with *BCR::ABL1*	Acquisition of *BCR::ABL1* through the course of AML
AML with *BCR::ABL1* according to OMS and ICC	YES	NO

**Table 6:** Genetic characteristics of AML with *BCR::ABL1* compared with AML and BC-CML.

**CML-specific fusion/mutations**		**AML**	**AML with** ***BCR::ABL1***	**Myeloid** **BC-CML**
*BCR::ABL1* fusion		rarely	**X**	**X**
	- as primary event		**X**	**X**
	- as secondary event	**X**		
Presence of BCR::ABL1 TKD mutations				**X**
**AML-specific fusions/mutations**		**AML**	**AML with** ***BCR::ABL1***	**Myeloid** **BC-CML**
Presence		**X**	**X**	
	- as primary event	**X**		
	- as secondary event		**X**	
**AML/BC-CML mRNA expression profile**		**AML**	**AML with** ***BCR::ABL1***	**Myeloid** **BC-CML**
AML-like profile		**X**	**X**	
*ID4* mRNA expression		**X**	**X**	
*CD25* mRNA overexpression				**X**

## Data Availability

The data that support the findings of this study are available from the corresponding author upon reasonable request.

## Supplementary Materials

The following supporting information can be downloaded at: https://www.mdpi.com/article/10.3390/ijms242015441/s1.
